# Advances in silicon-based, integrated tunable semiconductor lasers

**DOI:** 10.1515/nanoph-2022-0699

**Published:** 2023-01-18

**Authors:** Changjin Yang, Lei Liang, Li Qin, Hui Tang, Yuxin Lei, Peng Jia, Yongyi Chen, Yubing Wang, Yu Song, Cheng Qiu, Chuantao Zheng, Huan Zhao, Xin Li, Dabing Li, Lijun Wang

**Affiliations:** State Key Laboratory of Luminescence and Applications, Changchun Institute of Optics, Fine Mechanics and Physics, Chinese Academy of Sciences, Changchun 130033, China; Daheng College, University of Chinese Academy of Sciences, Beijing 100049, China; Peng Cheng Laboratory, No. 2, Xingke 1st Street, Shenzhen 518000, China; Jlight Semiconductor Technology Co., Ltd. No. 1588, Changde Road, ETDZ, Changchun 130102, Jilin, China; State Key Laboratory on Integrated Optoelectronics, College of Electronic Science and Engineering, Jilin University, Changchun 130012, China

**Keywords:** heterogeneous integration, hybrid integration, narrow linewidth, silicon nitride, silicon photonics, tunable semiconductor lasers

## Abstract

Tunable semiconductor lasers have many important applications such as wavelength division multiplexing, light detection and ranging, and gas detection. The increased interest in silicon photonics has led to the rapid development of miniaturized on-chip tunable semiconductor lasers. However, silicon has poor light-emitting properties. Therefore, realizing high-performance tunable semiconductor lasers requires the integration of light sources with silicon. In this study, we review silicon-based light source integration methods and the development of silicon-based integrated tunable semiconductor lasers. Considering that narrow-linewidth performance greatly expands the applications of tunable semiconductor lasers, methods for reducing the linewidth of tunable lasers are summarized. Finally, the development trends and prospects for silicon-based integrated light sources and silicon-based integrated tunable lasers are analyzed and discussed.

## 1 Introduction

Tunable semiconductor lasers are indispensable and important components in many fields because they are small, light, integrable, and tunable to different lasing wavelengths, which makes such lasers suitable for a broad range of functions. For example, to satisfy high-speed and capacity communication requirements, dense wavelength division multiplexing (DWDM) is currently one of the most effective technologies in core and optical access networks [1]. Tunable semiconductor lasers are vital light source devices in a DWDM system. For example, for a system with 96 channels and 50 GHz channel spacing, 38 nm wavelength tuning range is needed and the linewidth is in the order of 100 kHz [2]. Due to the lack of frequency sources, the demand for tunable semiconductor lasers in ultra-DWDM systems will continue to increase. Tunable semiconductor lasers are also indispensable in the field of sensing, and light detection and ranging (LIDAR), especially optical phased array-based LIDAR, is currently being actively studied. All-solid-state LIDAR requires a widely tunable semiconductor laser source that can be integrated into the same chip as an optical phased array, given that a wide tuning range ensures a large angle scan in the longitudinal direction [3, 4]. Tunable laser with output power of 20 mW, tuning range of 53 nm and linewidth of 100 kHz has been successful applied to an LIRDA system which achieves a scanning angle of 7°. A 15° scanning angle requires a laser wavelength tuning range of 100 nm. As for a 20° scanning angle, the tuning range should be greater than 140 nm [5]. Swept-source optical coherence tomography uses tunable lasers as light sources because they exhibit ultra-high-speed scanning, non-invasive imaging, high-resolution, and real-time imaging. This makes them extremely valuable in medical clinical diagnosis applications. The tunable lasers in the system are required to tune fast to realize a high imaging speed. Devices achieve over 70 nm wavelength tuning at over 30 mW of output power and show a high imaging speed at 800 kHz A-scan rates [6]. Moreover, tunable lasers can be used as a light source in gas detection to generate high-resolution characteristic absorption spectra of gases, thus improving the sensitivity of gas detection systems. In addition, tunable lasers have important applications in the fields of spectroscopy, terahertz communication, and optical clocks.

Like other optoelectronic devices, tunable lasers have known increasing development since their emergence. The production cost has been continuously reduced, which allowed large-scale production capacity. Integrated tunable semiconductor lasers have been receiving considerable attention as a research topic due to their small size, low cost, superior performance, and integrability. In addition, the rapid development of silicon optical technology provides new opportunities for tunable semiconductor lasers. Silicon, silicon dioxide, and silicon nitride are transparent in the 1300 and 1550 nm bands for standard communications and different material combinations can form high refractive index contrast to realize effective confinement of the optical mode field. This gives silicon-based optical waveguides superior performance to realize passive optical devices with different functions. In addition, they are compatible with the technologically mature complementary metal-oxide-semiconductor (CMOS) fabrication processes. This allows large-scale photonic device production at a low cost, providing a highly valued platform for optical interconnects. However, because silicon is an indirect-bandgap semiconductor, its luminescence performance is poor due to the simultaneous existence of free-carrier absorption, Auger recombination, and indirect recombination during the carrier transition process, resulting in very weak photon emission. The lack of an effective integrated light source has long restricted the development of silicon-based integrated photonic technology [7]. In contrast to silicon, III–V materials have excellent light-emitting properties. Combining III–V materials with silicon to form a silicon-based integrated tunable laser has become an effective solution to the lack of light sources for silicon-based integration platforms. Considerable efforts have been made in this area in recent years.

This paper is organized as follows. We start with a review of the methods and progress made with silicon-based integrated light sources and compare the different methods. Next, we present the status and progress of research on silicon-based integrated tunable semiconductor lasers with the integration methods already described. We then summarize and analyze the methods used for narrowing the spectral linewidth of silicon-based integrated tunable lasers. Finally, the development trends and prospects for silicon-based integrated light sources and tunable lasers are summarized.

## 2 Integration methods

An increasing number of researchers are focusing on integrating III–V gain media into silicon-based photonic platforms, owing to the excellent luminescent properties of III–V materials and the rapid development of silicon-based photonic technology. The main approaches can be divided into hybrid integration, heterogeneous integration, monolithic integration, and micro-transfer printing (μTP) [8–16]. In this section, we will elaborate on these categories.

### 2.1 Hybrid integration

Hybrid integration consists in combining separately prefabricated and optimized devices [17]. The existing combination methods include a flip chip and in-plane butt-joint coupling techniques.

#### 2.1.1 Flip-chip technology

**Figure 1:**
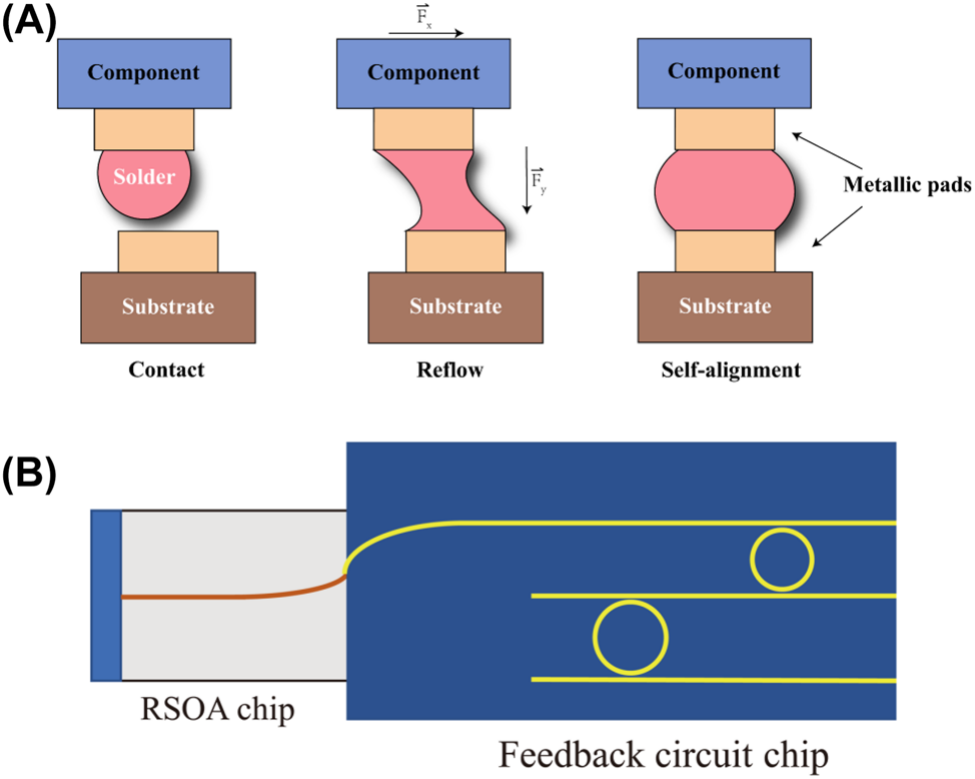
(A) Flip-chip self-alignment of the component and substrate during reflow [18]. (B) In-plane butt-joint coupling method.

A flip chip is a process whereby a chip is connected to a substrate or circuit board, with the solder bumps on the chip attached facing downward; hence it is called a flip chip. This process can be divided into four steps, starting with the formation of contact pads on both the chip and substrate, also known as under-bump metallization. These contact pads are generally deposited through sputtering, vapor deposition, or electroplating. With the metallization process, good electrical contact is realized, and a surface is formed for a molten solder alloy. The second step consists of growing solder bumps on the contact pad on the top surface of the chip; there are several growth methods, the most common being the electroplating process. When observed under a scanning electron microscope, the finished bumps show a uniform metallic sphere. Next, the chip is flipped and positioned precisely over the substrate to ensure the bumps are aligned with the contact pads on the substrate. The solder ball bumps are then remelted and soldered to the under-bump metallization pads of the substrate. At this stage, the chip self-aligns with the substrate through the surface tension of the molten solder. This process is shown in Figure 1A [18]. Finally, an electrically insulating adhesive fills the gap between the chip and the substrate to isolate the external environmental influence and provide a solid connection. Furthermore, the differences in the coefficients of thermal expansion get moderated. Currently, the choices of underfilling adhesives have increased, and breakthroughs have been achieved for the underfilling technology between the substrate and the chip [19–21]. However, the gap is too wide for photonic circuits, making the optical coupling between components difficult and inefficient. To solve this problem, thermal compression bonding technology has been widely used [22–25] recently to reduce the gap.

The alignment accuracy is another challenge; the improved alignment accuracies are around ±1 μm, which is not small enough to obtain high coupling efficiencies [26]. To ensure alignment accuracy during the flip-chip process, marker-assisted alignment can be performed on the substrate and device surfaces, and the accuracy can be further improved by active alignment. In 2019, Theurer et al. [27] successfully achieved the precise integration of a distributed-feedback (DFB) laser to a SiN photonic platform. They detected the signal at the output of the SiN chip to ensure horizontal alignment accuracy while marking the alignment of the laser and the chip. In 2020, the same group used the optical backward scattering reflection method to monitor the gap between the laser chip and the SiN chip by optimizing the active alignment process and achieved 2.1 dB of average insertion loss, further improving the alignment accuracy [28].

#### 2.1.2 In-plane butt-joint coupling

In-plane butt-joint coupling techniques refer to the direct alignment coupling of one end of one device to the other end of another device in the same plane, as shown in Figure 1B. III–V semiconductor lasers integrated by butt-joint coupling can achieve excellent performance such as a low-threshold current, low relative intensity noise, and high output power. However, there are still many challenges in this coupling process. First, the problem of optical mode mismatch needs to be solved. Usually, the optical modes of the two chips are not matched. For example, the mode size of a semiconductor optical amplifier is approximately 1 μm in the vertical direction and a few micrometers in the lateral direction, while the typical size of a silicon waveguide is of the order of hundreds of nanometers. When these two devices are directly coupled, the mode mismatch leads to extremely low coupling efficiency. To achieve optical mode matching, researchers have proposed using either a spot-size converter [29, 30] or a grating coupler [31–33]. The other major challenge is the need for highly accurate alignment during component assembly. Similar to flip-chip integration, the alignment accuracy can be optimized using active alignment.

Hybrid integration is currently one of the most mature integration technologies. Its most prominent advantage is that the manufacturing and optimization of each discrete device can be completed before the integration; thus, the devices do not interfere with each other during the integration process. However, both flip chip and in-plane butt-joint coupling technologies have very high alignment accuracy requirements; thereby, it is difficult to achieve industrial mass production and high-density integration.

### 2.2 Heterogeneous integration

**Figure 2:**
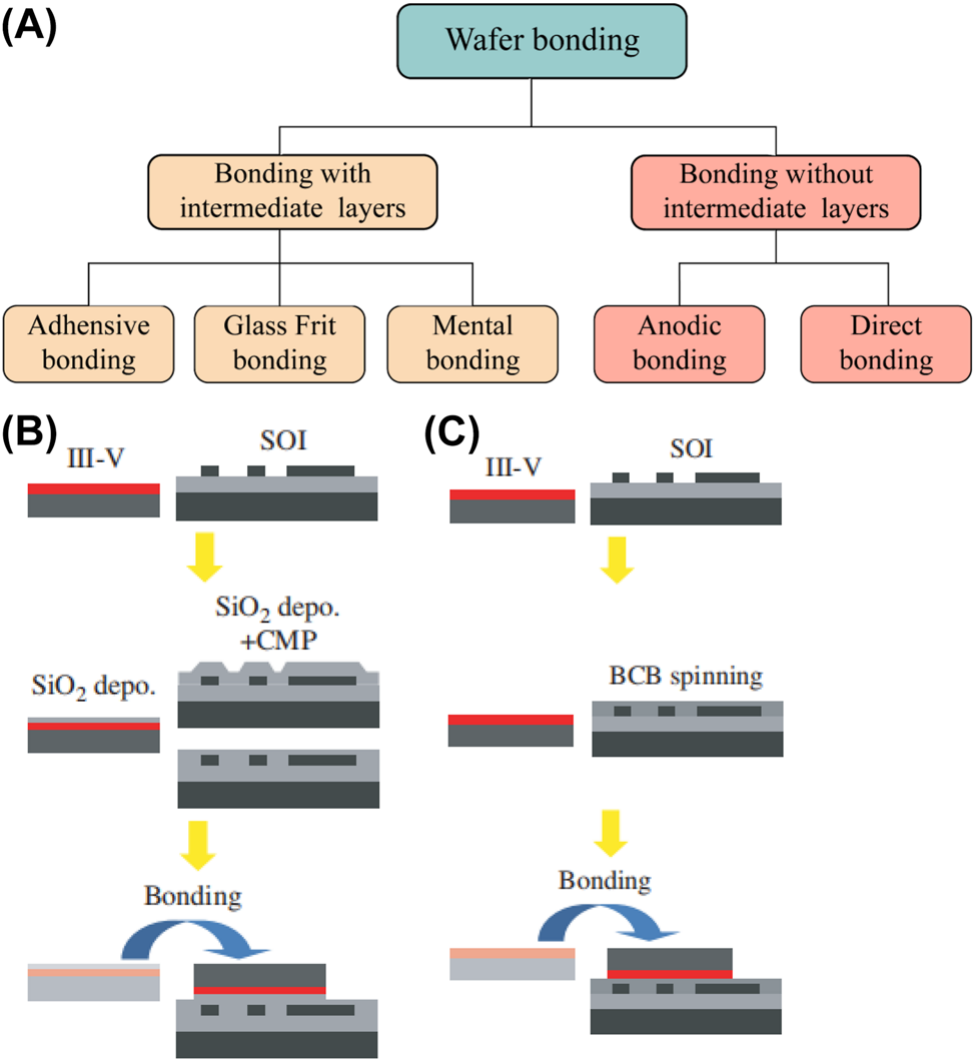
(A) Wafer bonding technology classification. (B) SiO_2_ direct bonding. (C) Divinylsiloxane-bis-benzocyclobutene (DVS-BCB) adhesive bonding [34].

Heterogeneous integration generally refers to the process of transferring non-silicon, unprocessed thin-film materials onto silicon substrates using wafer bonding techniques and then planar optical waveguide techniques to achieve specific optical functions. Wafer bonding is the process of connecting two or more substrates or wafers via physical or chemical interactions. Different wafer surfaces are combined either through their atoms mutually reacting with each other or by an adhesive intermediate layer. Wafer bonding has become an important technology for 3D integration and packaging, and manufacturing silicon-on-insulator (SOI) wafers, micro-electromechanical systems, micro-opto-electromechanical systems, and photonic systems. Today, a wide variety of bonding technologies is available. One of the classification methods is based on the presence or absence of an intermediate layer. As shown in Figure 2A, the techniques without an intermediate layer are classified as anodic or direct bonding, whereas those with an intermediate layer are classified as adhesive, glass frit, or metal bonding.

In the process of anodic bonding, the temperature and voltage are high, and there can be problems such as contamination with charged ions. In glass frit bonding, the thickness of the intermediate adhesive layer is generally >5 μm. This excessively thick intermediate layer makes the evanescent-wave coupling difficult. In the metal bonding process, the light is strongly absorbed by the metal. Hence, none of these bonding techniques are suitable for the heterogeneous integration of III–V materials with SOI platforms. Therefore, both direct and adhesive bonding techniques have been widely studied and applied to heterogeneous integration.

#### 2.2.1 Direct bonding

Direct bonding, first proposed by Lasky et al. in 1986 [35], directly connects the surfaces of two mirror-polished semiconductor wafers by mechanical or electric field action without any intermediate layer. The two surfaces are in intimate contact through van der Waals forces or hydrogen bonding. However, this state is not automatically achievable because factors such as surface roughness, wafer unevenness, and the presence of microscopic particles prevent such close contact between the surfaces; thereby, after the two wafers are placed in contact, pressure is usually applied to the wafer center (or side) wherein the surfaces are in close contact, and localized bonding is achieved at the pressure point. Then, the wafer surfaces enter into close contact due to the attraction between the wafers, and the bonding wave spreads from the pressure point to the periphery of the wafer, achieving close contact in a few seconds. However, this adhesion is much weaker than covalent bonding, and subsequent high-temperature annealing is required. The annealing temperature is usually above 800 °C and can be reduced by plasma activation or other special wafer surface processing. A SiO_2_ direct bonding process is illustrated in Figure 2B [34].

In 2000, Pasquariello et al. [36] described an oxygen plasma-activated wafer bonding process with annealing temperatures as low as 200 °C. In 2008, Liang et al. [37] activated the silica surface of SOI and InP wafers by oxygen plasma at an annealing temperature of 300 °C. This optimized direct bonding process enabled the demonstration of several III–V/Si integrated lasers, including micro-disk [38], Fabry–Perot (FP) [39], DFB [40], and several different tunable lasers [41]. However, direct bonding imposes high requirements on the contact surface, which is usually cleaned before bonding using the semiconductor industry standard wet cleaning process [42] known as Radio Corporation of America cleaning. There are two hydrogen peroxide-based steps known as RCA1 (NH_4_OH:H_2_O_2_:H_2_O = 1:1:5), and RCA2 (HCl:H_2_O_2_:H_2_O = 1:1:6). The first step is designed to remove organic contaminants, especially hydrocarbons, using NH_4_OH and H_2_O_2_. The second step is mainly used to remove metallic (ionic) contaminants. The whole bonding process should be performed in an ultra-clean environment and must be inspected in real-time using infrared imaging or c-type scanning acoustic microscopy.

#### 2.2.2 Adhesive bonding

Adhesive bonding is conducted by spin-coating an adhesive onto the surface of one or the two wafers, aligning them face-to-face, and applying pressure to bring the wafer surfaces into close contact. The adhesive is then heated or UV-irradiated to convert from a liquid or viscoelastic state to a solid state. A general process can be described as follows: (1) cleaning and dehydrating; (2) depositing adhesion promoter on the wafer surface, usually by vapor deposition, thereby enhancing the adhesion of the wafer surface to the adhesive; (3) spin-coating the adhesive and initiating the formation of a pattern; (4) pre-treating the adhesive to remove solvents and volatile substances from the adhesive; (5) placing the wafer into the bonding apparatus under vacuum and applying a pressure to force the bonding surfaces into close contact; (6) heating to achieve the bonding. Among the different types of adhesives, the most commonly used is divinylsiloxane-bis-benzocyclobutene (DVS-BCB) [43, 44]. Figure 2C illustrates an adhesive bonding process using DVS-BCB [31]. The advantages of DVS-BCB can be summarized as follows: (1) low optical loss at communication wavelengths of approximately 1310 and 1550 nm, (2) low deformation shrinkage after curing, and (3) excellent planarization properties.

However, adhesive bonding still raises many challenges: (1) The adhesives have relatively low thermal conductivity, which increases the thermal impedance of the fabricated devices; (2) Some adhesives react and produce by-products or may shrink; and (3) The process exhibits poor sealing and low alignment accuracy. Compared with direct bonding, adhesive bonding has several outstanding advantages: (1) relatively low requirements in terms of wafer surface roughness and defects, and a relatively high tolerance for small particles; (2) relatively low processing temperature (<300 °C); and (3) theoretical bonding compatibility with any type of wafer material. These advantages make it more promising for large-scale industrial production. In 2005, BCB adhesive bonding was used to realize micro-ring resonators (MRRs), LEDs, and pulsed lasers [45]. In 2012, a BCB adhesive bonding-based 1310 nm emission III–V/silicon DFB laser was realized. The output power was 2.85 mW at 10 °C, the threshold current was 20 mA, and the side mode suppression ratio (SMSR) was 45 dB [46]. In addition, Keyvaninia et al. studied the BCB layer-thickness reduction technology and achieved an ultra-thin BCB bonding with a thickness of approximately 35 nm [47].

### 2.3 Monolithic integration

Monolithic integration of III–V semiconductor materials on silicon substrates through heterogeneous epitaxial growth is considered the optimal solution for silicon optical integration [48], both in terms of integration scale and economic cost. Epitaxy allows the selective growth of the required III–V material at wafer size without the need for complex bonding processes and precise alignment requirements. Therefore, the monolithic integration solution has recently developed considerably; however, it still lags behind the hybrid and heterogeneous integration methods in terms of application.

Threading defects, misfit dislocations, and anti-phase boundaries are generated during the epitaxial growth of III–V semiconductor materials on silicon (001) substrates. This is due to the lattice constant, thermal expansion coefficient, and polarity mismatches between different materials, which significantly affect the growth quality of materials and device performance. For dislocation defects, researchers have recently proposed the insertion of III–V quantum dots into the active region as defect filter layers. Simultaneously, the quantum dots act as advanced gain material [49–51]. In 2014, Liu et al. used quantum dots as gain by growing multiple strain-layered superlattices as dislocation filter layers. The threading dislocation density was significantly reduced [51].

**Figure 3:**
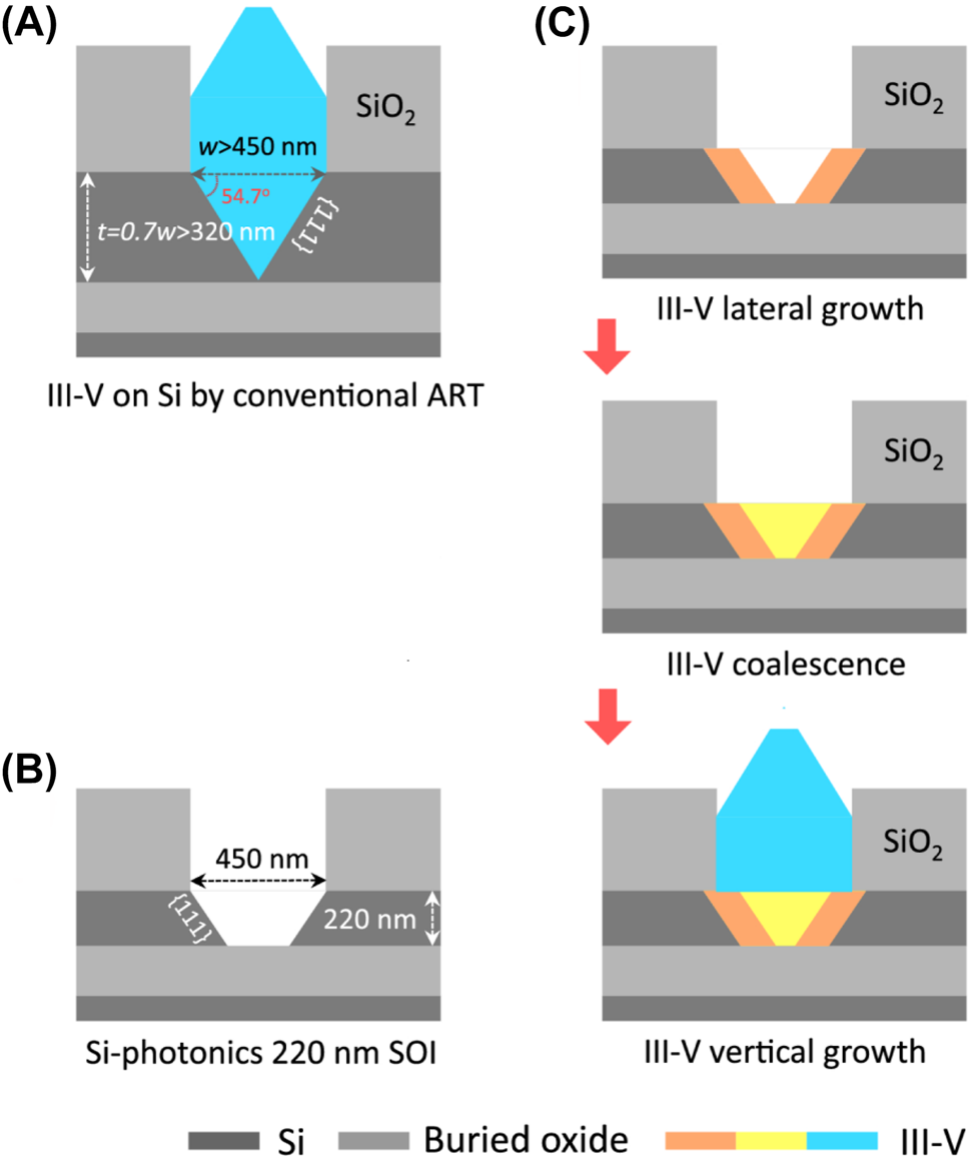
(A) Conventional aspect ratio trapping method with III–V epitaxial growth on a silicon V-groove. (B) Predefined trapezoidal groove on SOI wafer. (C) Designed growth sequence of bufferless III–V on Si-photonics 220 nm SOI platforms [55].

However, this approach is not useful for the anti-phase boundaries challenge. Epitaxial growth on an offcut silicon substrate (usually 4° offset in the [52] direction) is a common solution; however, it is incompatible with the CMOS industry, and therefore is not suitable for large-scale production. Another approach is to grow a buffer layer, generally using GaAs because of their small lattice mismatch with silicon (4.1%) [53]. However, for the integration of III–V materials and silicon waveguides, the use of thick buffer layers prevents light coupling into the silicon waveguides. Because of this shortcoming, researchers proposed a technique for selective epitaxial growth with almost no buffer layers, using the aspect ratio trapping method to direct and confine defects to the sidewalls, ensuring a defect-free epitaxial layer. In 2015, Wang et al. successfully demonstrated a 925 nm optically pumped InP DFB nanowire laser using selective growth of InP in a predefined silicon V-groove [54]. Figure 3A shows a conventional aspect ratio trapping method with III–V epitaxial growth on a silicon V-groove. In 2020, Han et al., designed and fabricated InP and InP/InGaAs lasers by selective epitaxial growth of high-quality III–V materials in a predefined trapezoidal groove on an SOI wafer. They achieved room temperature pulsed lasing at 900 and 1500 nm [55]. Figure 3B and C illustrate a predefined trapezoidal groove on an SOI wafer and the schematics of the designed growth sequence of bufferless III–V on Si-photonics 220 nm SOI platforms, respectively [55].

In addition, epitaxial lateral overgrowth is widely used to avoid thick buffer layers [56–58]; a III–V seed layer is deposited on the silicon substrate surface by metalorganic chemical vapor deposition or molecular beam epitaxy. Then, a mask with appropriately spaced openings is fabricated on the deposited seed layer so that the material matching the lattice of the seed layer can grow. After the material grows to the edge of the mask, an epitaxial structure grows laterally along the mask. To prevent threading dislocations from entering the growing epitaxial lateral overgrowth layer, the width of the mask openings must be smaller than the thickness of the mask due to the “necking effect”.

### 2.4 Micro-transfer printing technology

Micro-transfer printing (μTP) technology is a new integration technique developed by Menard et al. in 2004 [59]. Micrometer-level films are transferred on a large scale from the original substrate to the target substrate, ensuring high-throughput and high-accuracy integration with a very short process cycle time, typically 30–45 s. This new integration technology lies between heterogeneous and hybrid integration, with the advantages of both. Applying this technology to silicon integration reduces the cost, enables large-scale integration, and guarantees high-quality devices.

**Figure 4:**
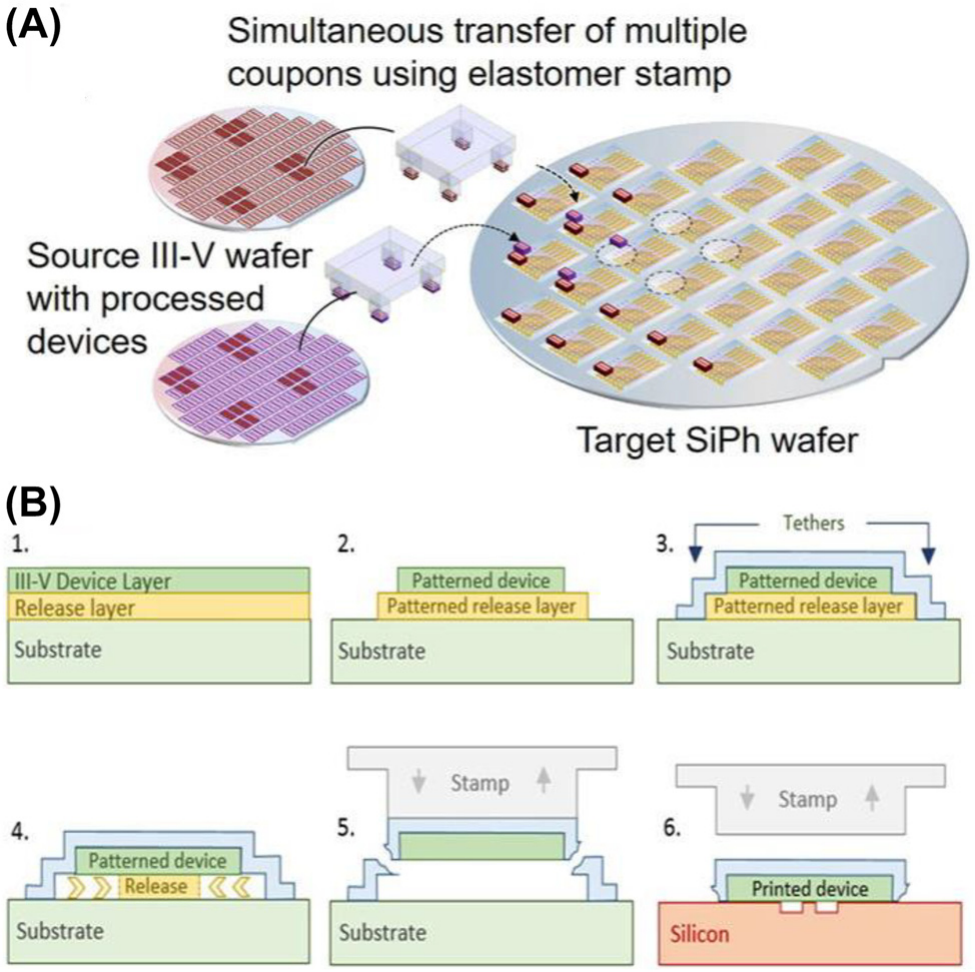
(A) μTP-based integration on 200 or 300 mm Si photonic wafers in a parallel manner [60]. (B) Prefabrication of III–V devices on the native substrate and μTP integration process [61].

As illustrated in Figure 4A, the μTP is achieved using a flexible elastomer polydimethylsiloxane (PDMS) stamp tool with an array of columns (or individual columns). The columns match the size and spacing of the devices on the original substrate and are used for picking up these devices and transferring them to the target substrate [60].

A typical III–V device transfer to a silicon substrate [60, 61] is shown in Figure 4B. There is a thin release layer between the sample and the substrate of the III–V device, and for the InP material system, InGaAs/InAlAs is generally chosen as the release layer. First, the coupons are patterned on the original substrate, and then they are protected by encapsulating photoresist on the surface, which leaves local openings to access the release layer. Next, the release layer is etched laterally using a low-temperature FeCl_3_ solution (for InP-based material) with an etch selection ratio greater than 1000. After etching, the PDMS stamp is quickly lifted to pick up the coupons, and the tethers break on both sides. Afterward, the coupons are brought into contact with a BCB adhesive-coated substrate, and the adhesive force makes the coupons and the PDMS stamp separate to complete the transfer process.

In 2012, Justice et al. successfully demonstrated an FP cavity laser using μTP technology, achieving continuous low-threshold lasing at a wavelength of 824 nm and temperature of 100 °C [62]. Unlike in Figure 4, they first transferred the coupons to the substrate, and then patterned and opened the electrodes on the target substrate. The μTP technology has been successfully implemented on silicon–photonic integrated circuits (PICs) for several different device integrations, including photodiodes [63] and lasers, such as FP [64], distributed Bragg reflector (DBR) [65], DFB [66], vertical-cavity surface-emitting [67], and tunable lasers [68]. More details on μTP technology can be found in the review by Zang et al. [60].

The μTP technology has very attractive prospects, offering a new optimized solution for silicon photonics integration. However, there are also some challenges. First, when the coupon is released from the source substrate before pick up, due to the increased length, the stress in the layer stack might bend or twist the free-hanging coupon, which in turn degrades the bonding quality. Second, it is important to achieve precise alignment. The tolerance for misalignment (±1.5 μm 3*σ*) is achieved when printing in large arrays through a pattern recognition function. To further improve the alignment accuracy, more effort is needed.

### 2.5 Summary of integration methods

Six aspects of the four integration technologies are compared in Table 1.

**Table 1:** Comparison of different integration methods.

Technology	Integrationdensity	CMOS process compatibility	Alignmentaccuracy	Productionvolume	Cost	Technology maturity
Hybrid integration	Low	Back-end compatibility	Medium	Low	High	Mature
Heterogeneous integration	Medium	Back-end compatibility	High	High	Medium	More mature
Monolithic integration	High	Possible front-end compatibility	Medium	High	Low	Research and development
Micro-transfer printing	High	Back-end compatibility	High	High	Low	Research and development

Although hybrid integration is a very mature technology, its high cost and low production volume make it unsuitable for high-density integration and wafer-level production. Heterogeneous integration based on bonding processes is still the optimal solution. Adhesive-based bonding, notably, shows unique advantages. Monolithic integration and μTP are still in the research and development stage; however, due to their excellent technical characteristics, they show good development prospects, attracting a large number of researchers. They are expected to become the mainstream solution for silicon photonics integration in the near future.

## 3 Developments of tunable semiconductor lasers

The development of tunable semiconductor lasers has led to breakthroughs and advances in tuning range, tuning speed, and device size. With the development of silicon photonics, integration-capable silicon-based tunable semiconductor lasers have become a focus for research due to their low cost, small size, and good performance.

Currently, the most common tuning methods are thermal and electrical tuning, which are based on the principle of changing the waveguide refraction through a thermal-optical or electro-optical effect. Thermal tuning is slower, but introduces very little loss, whereas in electrical tuning the extra carriers have an absorption effect on photons, leading to high losses. Different tuning methods can be chosen for different applications. To avoid high losses, thermal tuning is the more widely used method.

To achieve a wide tuning range, silicon-based integrated tunable semiconductor lasers based on the Vernier effect of multiple filters have been widely studied. They include the MRR and sampled grating DBR (SGDBR) types. By combining lasers operating in different wavelength ranges, the entire laser array can operate collectively to achieve a wide tuning range.

In this section, we review the research progress of silicon-based tunable semiconductor lasers based on the integration techniques described above.

### 3.1 Hybrid integrated lasers

Hybrid integration is the most mature and most widely used today. Organizations including NEC [69–71], Northeastern University [72–75], Oracle Corporation [76, 77], Nokia Bell Labs [78, 79], University of Twente [80, 81], and Shanghai Jiao Tong University [82, 83] have successfully demonstrated silicon-based tunable semiconductor lasers based on hybrid integration.

In 2006, Ishizaka and Yamazaki [84] connected a semiconductor optical amplifier (SOA) to a PIC platform using in-plane butt-joint coupling. The PIC consisted of two cascaded MRRs with slightly different sizes. The Pt heater was placed above one of the MRR waveguides with SiON core material. The PIC platform size was approximately 7 × 3 mm. With a heater power consumption of approximately 870 mW, a tuning range of approximately 45.2 nm in the C- and L-bands was achieved using the Vernier effect. The fiber coupling output power was only 4.7 dBm at 167.5 mA input current. The low coupling output power is attributed to an insufficient coupling between the SOA and the external cavity, and between the SOA and the single-mode fiber.

In 2009, Chu, Fujioka, and Ishizaka [69] improved the work by Ishizaka and Yamazaki [81] using an SOI waveguide instead of a SiON waveguide. This change enabled the bent radius of the micro-ring to be reduced, and a larger free spectral range, stable single-mode output, and high SMSR were obtained. Moreover, the large thermo-optical coefficient of silicon allows for a smaller power consumption of the heater during tuning. By heating the heaters on both micro-rings, the tuning range reached 38 nm with a power consumption of only 26 mW. To minimize the optical modes mismatch between the SOA and SOI waveguides, a spot-size converter was used to reduce the transmission and reflection losses during coupling.

In 2015, Kobayashi et al. [71] realized a hybrid integrated tunable laser using passive alignment technology to achieve over 100 mW fiber output power with a tuning range of 65 nm. The device consisted of a micro-ring filter chip, gain chip, and booster SOA chip. There were corresponding alignment marks both on the chip surface and the bottom of the platform. Using infrared light to illuminate the contours of the alignment marks, the image was recognized by the camera, and the center position of the marks was automatically calculated. Horizontal and vertical passive alignment were then successfully realized, achieving high optical coupling between these chips.

By optimizing the cladding structure and improving the waveguide annealing process, the authors reduced the roughness of the waveguide and achieved an ultra-low-loss silicon waveguide with a loss of <0.5 dB/cm in the whole C-band. The linewidth of the laser was considerably reduced, and the laser achieved excellent performance with a linewidth <15 kHz in the C-band. The use of a booster SOA to control the output power effectively reduced the photon density in the external cavity, thereby reducing the impact of nonlinear effects such as four-wave mixing and two-photon absorption (TPA), which ensured a stable and high-power output.

In 2015, Kita, Tang, and Yamada [73] introduced an asymmetric Mach–Zendel interferometer (MZI) into a parallel double micro-ring structure; a 99 nm tuning range was achieved using a slightly asymmetric MZI with a free spectral range approximately twice that of the two micro-rings. When the lengths of the two arms of the MZI were highly asymmetric (the difference was 1578 μm), the laser mode gain difference was larger, and the linewidth was effectively compressed. The laser achieved a 42.7 nm tuning range with a linewidth of only 12 kHz. In 2017, de Valicourt et al. [78] achieved tuning range expansion using an MZI. They noted that the tunable range of a single micro-ring was only 16 nm, and when an asymmetric MZI was added, a 31 nm tuning range was achieved, which is comparable to the 32 nm tuning range of the double ring.

**Figure 5:**
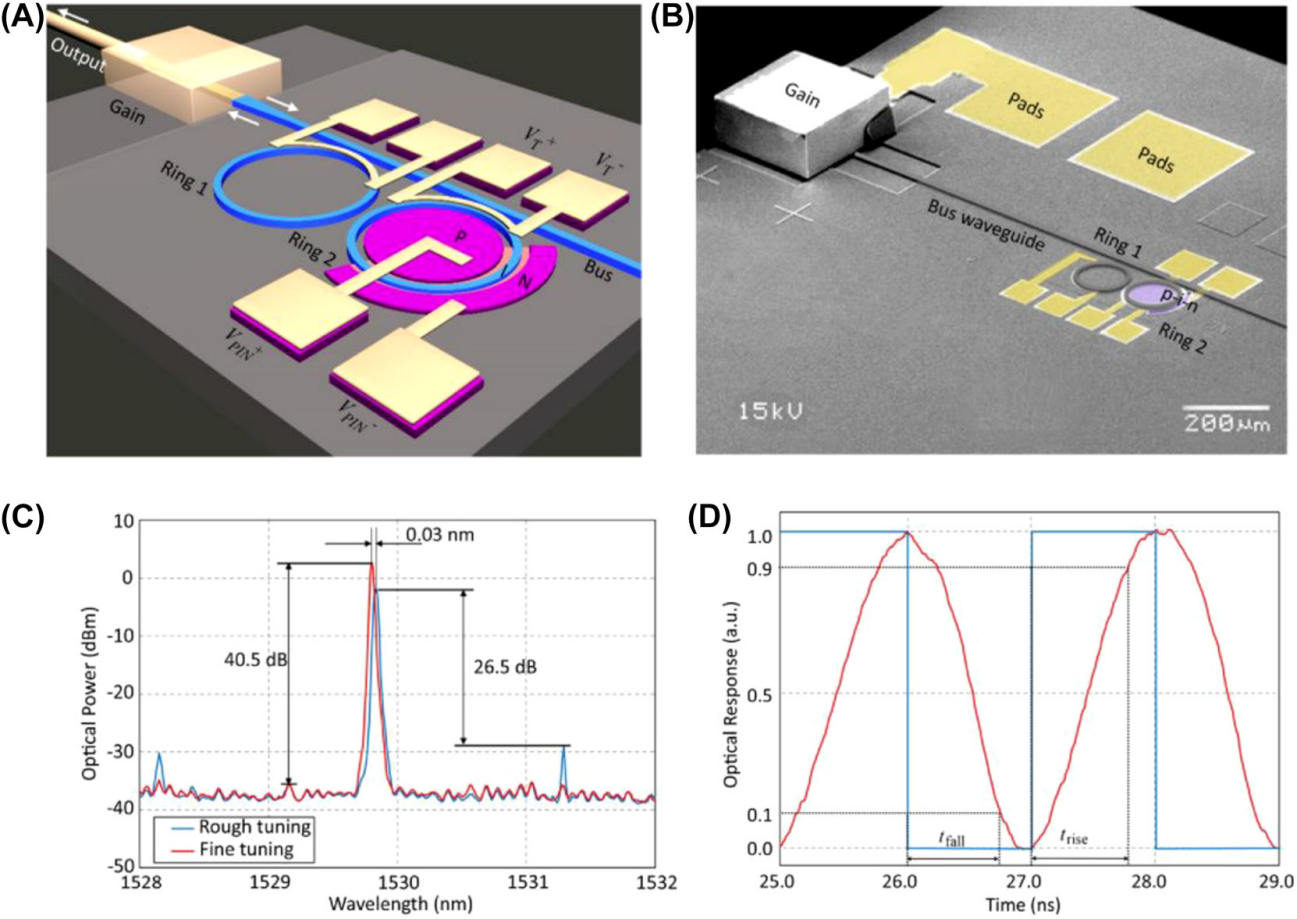
Integrated silicon–photonic tunable laser: (A) schematic, (B) scanning electron microscopy image, (C) accuracy of wavelength tuning due to free-carrier injection, and (D) response speed of free-carrier dispersion effect [85].

Tunable lasers with high tuning speed and accuracy are required in some applications such as high-speed optical networks, in addition to achieving a wide range of tuning functions. In 2015, Ren et al. [85] designed and demonstrated a silicon-based external cavity tunable laser integrated by the flip-chip method. The schematic and scanning electron microscopy images of the laser are shown in Figure 5A and B, respectively. The effective optical length of the coupled ring reflector was controlled by complementary thermo-optical and free-carrier dispersion effects.

By heating the two rings, ring 1 was used to achieve continuous wavelength tuning, and ring 2 was used to control the lasing wavelength. In addition, there was a p-i-n junction on ring 2 to achieve a fast-fine-tuning function through electrical injection. This enabled high-resolution wavelength fine-tuning while maintaining a wide wavelength tuning range. The experimental results in Figure 5C and D shows that the tunable laser has high external cavity efficiency (22%), high wavelength tuning accuracy (2 pm), and an SMSR > 40 dB in the tuning range of 41.6 nm. When the p-i-n junction operates at a bias voltage of 1.0 V and modulation voltage of 1.6 V, the modulation frequency was set to 0.5 GHz. The rise and fall times were 0.78 ns and 0.72 ns, respectively, which was 103 times faster than thermal tuning.

### 3.2 Heterogeneous integrated lasers

Compared to hybrid integration, heterogeneous integration offers fundamental improvements in manufacturing accuracy, integration density, and production volume and efficiency, and has rapidly become one of the most popular on-chip source integration solutions after more than a decade of development [12, 86]. Currently, the main research institutes for heterogeneous integration based on silicon-based tunable semiconductor lasers include UCSB [87–95] and Ghent University [96, 97].

In 2008 Sysak et al. [98] realized an array of four silicon SGDBR lasers with a tuning range >13 nm, each consisting of a 600 µm back-end absorb region, 650 µm rear reflector, 80 µm phase region, 550 µm gain region, 270 µm front reflector, and 100 µm long tapered region. The rear mirror had fourteen 7.6 µm-wide gratings, each with a sampling period of 46.4 µm, while the front mirror had five 5.2 µm-wide gratings, each with a sampling period of 52.4 µm. The III–V gain section was heterogeneously integrated with an SOI waveguide via low-temperature plasma-assisted bonding.

In 2016 Duprez et al. [41] reported a tunable III–V/Si-SGDBR laser operating in the O-band. By designing different opto-geometrical parameters, they concluded that more tuning power was needed for a shorter sampling filling factor of the mirror. The smaller size difference between each mirror sampling period in the cavity allowed for more peak selection in the gain region, which resulted in a wide tuning range. Laser test results showed that in silicon waveguides, the output power was as high as 7.5 mW, reaching a continuous tuning range of 35 nm. The laser operated in single mode over the entire spectral range with an SMSR > 35 dB.

In 2016 Dhoore et al. [99] reported a tunable sampled grating DFB laser heterogeneously integrated on an SOI wafer. Wavelength tuning was achieved by changing the current injected into the two-stage DFB design. Compared with the DBR laser, the wavelength control was quite simple and there was no additional phase section to be electrically tuned. The laser achieved a wavelength tuning range of more than 55 nm in discrete wavelength steps of 5 nm, with an SMSR > 33 dB for all wavelength channels. Due to the high thermal resistance of the device, a wider continuous tuning range was not achievable. This problem is expected to be solved by redesigning the sampled grating and improving the thermal performance.

In addition, heterogeneous integrated tunable lasers based on the MRR have been extensively studied.

In 2013, Hulme, Doylend, and Bowers [87] designed a racetrack Vernier double-ring structure of a tunable laser with two Vernier rings sandwiched between two silicon waveguides and an InP gain region heterogeneously integrated with the silicon waveguide by bonding. In the bonding area, both the gain section and silicon waveguide adopted a tapered structure, which was beneficial to the evanescent coupling of the optical field mode between the III–V waveguide and silicon waveguide. The laser had a tuning range >40 nm, an SMSR > 35 dB, a maximum continuous-wave on-chip output power of 3.3 mW, and a central wavelength linewidth of 338 kHz.

**Figure 6:**
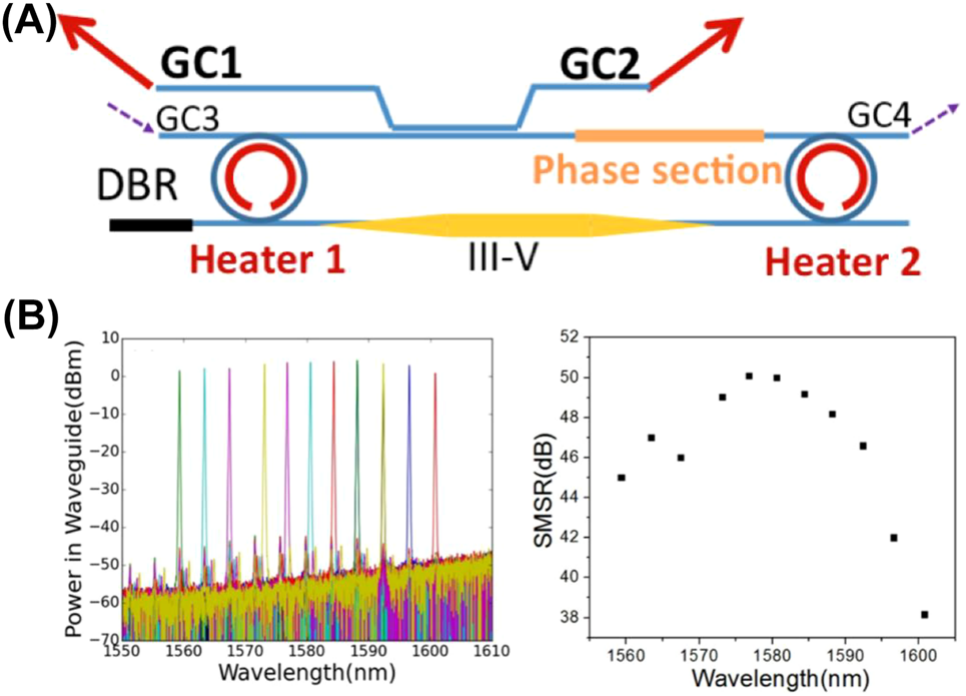
Unidirectional, heterogeneous integrated widely tunable laser: (A) schematic, and (B) output spectra and SMSR as functions of wavelength.

Komljenovic et al. [90] pointed out that although the racetrack structure is easy to tune and can achieve higher power output and SMSR, it is easy to generate bidirectional lasing, which results in the competition of two traveling waves because the whole structure is a loop. It is difficult to guarantee single-mode operation. In 2017, Zhang et al. [97] introduced a DBR reflector based on the above structure at one end to realize unidirectional lasing, as depicted in Figure 6A. The laser achieved a 40 nm tuning range with an SMSR > 35 dB, which can be seen in Figure 6B. They pointed out that by strengthening the feedback of the Bragg grating, the bidirectional lasing behavior can be suppressed, and the linewidth of the laser can be reduced.

In 2018, Tran et al. [92] proposed a new multi-ring mirror design. The rear cavity surface of the resonator comprised three cascaded micro-rings. Two of the rings had radii of 60 μm and 65 μm, and both rings had the same power coupling coefficient with the bus waveguide. The third ring had a larger radius (100 μm) and a wider waveguide width, and the coupling coefficient with the bus waveguide was only half of the former. The results showed that an average SMSR > 55 dB and a narrow linewidth of 17.5 kHz were achieved in the 30 nm tuning range.

### 3.3 Monolithic and μTP integrated lasers

Monolithic integration and μTP technology are still in the research and development stage; nevertheless, they have recently shown progress in tunable lasers [54, 68, 100–102].

**Figure 7:**
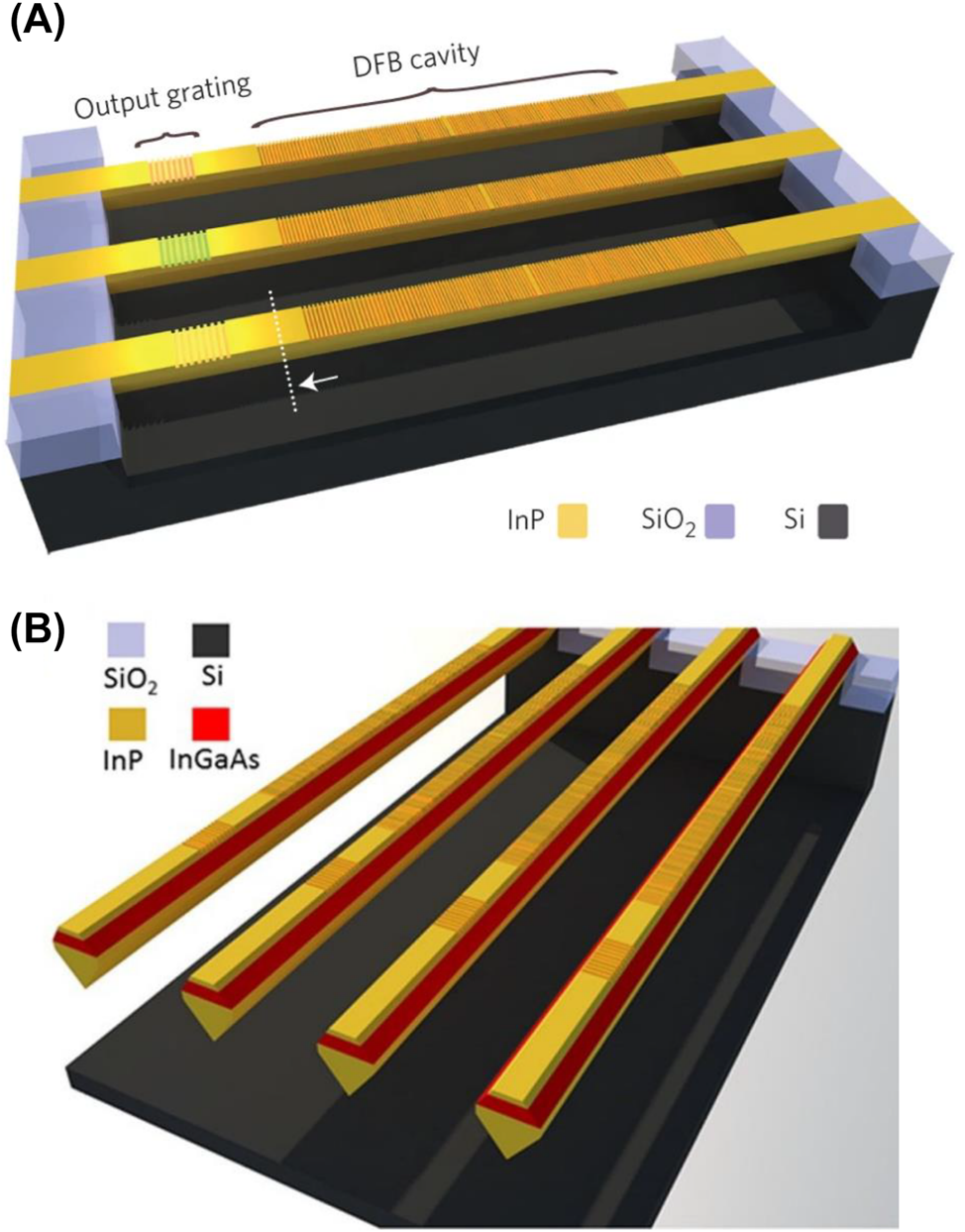
(A) Monolithically integrated InP DFB lasers on silicon [54]. (B) Monolithically integrated InGaAs/InP DFB lasers on silicon [100].

In 2015, Wang et al. [54] used selective epitaxial growth to grow a millimeter-long high-quality InP waveguide on a (001) silicon substrate at room temperature. A first-order grating was defined on the InP waveguide with an inserted *λ*/4 phase-shift section. To facilitate the characterization of the device, a second-order output grating was defined at 30 μm from the DFB cavity. Since InP, which has a higher energy bandgap than Si, was grown directly on Si, all light generated in the III–V material was absorbed directly in the Si substrate. Therefore, the Si substrate under the InP-based laser device was intentionally etched, presenting a suspended state. An array of optically pumped DFB lasers was fabricated, and the schematic is shown in Figure 7. By changing the length of the phase-shift section, the operating wavelength of the laser was changed.

In 2016, Tian et al. [100] used an InP-on-Si structure as a substrate for the subsequent growth of high-quality III–V epitaxial materials. An optically pumped DFB laser emission, operating at room temperature in the 1300 nm wavelength range, was successfully demonstrated by growing InP/InGaAs/InP heterostructures on the aforementioned substrate. The DFB laser was defined by etching the first-order grating on the top surface of the device, enabling stable single-mode operation with an SMSR > 25 dB. Compared to Wang et al. [54], a 65% reduction in threshold was observed. To demonstrate the scalability potential of this integrated platform, an array of 10 DFB lasers with grating periods ranging from 335–362 nm was fabricated, keeping the duty cycle and etch depth at 50% and 20 nm, respectively. A tuning range of almost 40 nm was achieved with a tuning step of 3 nm. However, the suspended structure could not realize current injection and effective heat dissipation.

In 2018, Wang et al. [101] Reported, for the first time, an array of electrically pumped continuous single-mode excitation DFB lasers grown epitaxially on silicon using InAs/GaAs quantum-dot gain materials at room temperature. Each laser had a threshold current as low as 12 mA, SMSR > 50 dB, and an array of six DFB lasers with a wavelength coverage of up to 100 nm and a channel spacing of 20 ± 0.2 nm by varying the grating period. This was a good match with a standard coarse wavelength division multiplexing grid.

In 2019, Wan et al. [102] demonstrated a tunable quantum-dot single-wavelength laser grown directly on silicon. The tunable laser consisted of two active ring resonators coupled to an FP cavity via two half-wave couplers and achieved a 16 nm tuning range (without thermoelectric cooler temperature control) with an SMSR > 45 dB under continuous-wave electrical injection at room temperature. They noted that the tuning range could be increased by reducing the perimeter difference between the two ring resonators or by changing the thermoelectric cooler temperature.

**Figure 8:**
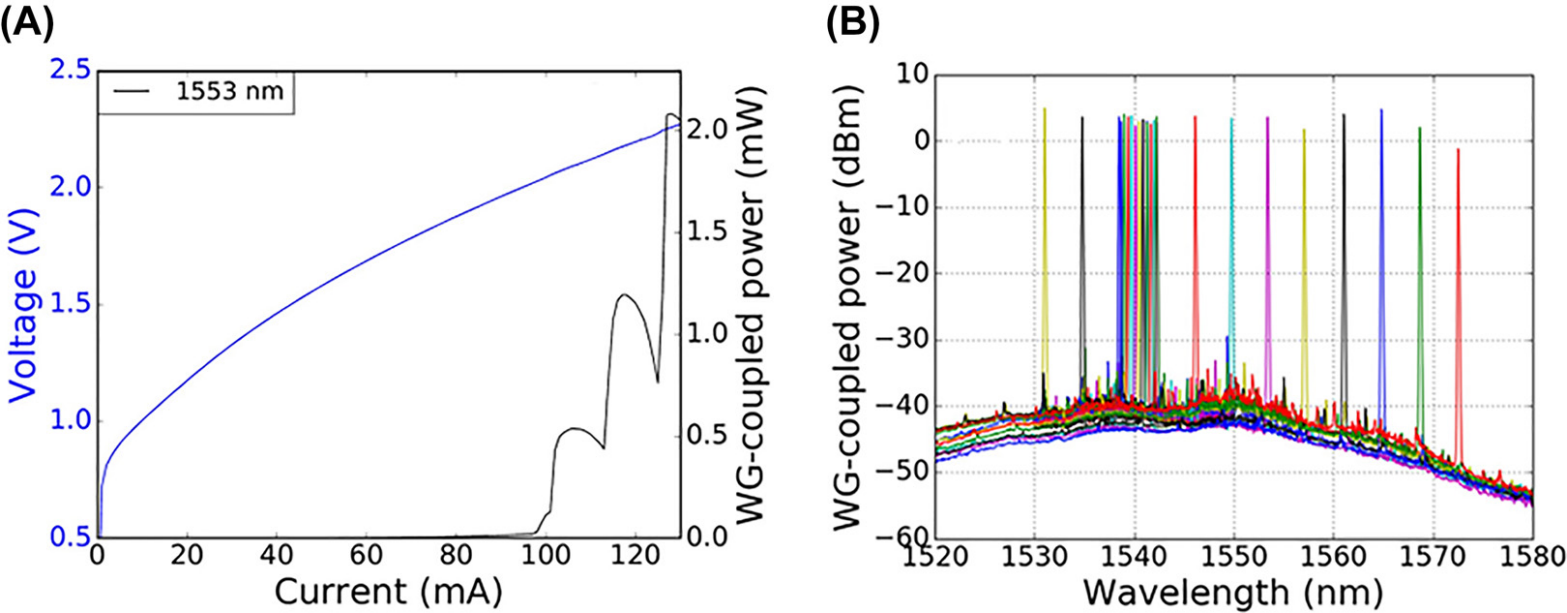
(A) L-I-V plot with a threshold current of 100 mA. (B) Wavelength tuning with a 40 nm tuning range [68].

In 2022, Zhang et al. [68] demonstrated the first III–V/Si tunable laser fabricated by μTP technology, achieving a 40 nm tuning range with a threshold current of 100 mA, as depicted in Figure 8A and B. The whole process included removing the back-end stack to form a recess to integrate the SOA by a combination of reactive ion etching and buffered HF wet etching. Then, a thin DVS-BCB layer was spray-coated on the chip. After soft baking at 150 °C for 15 min, the samples were mounted on an X-Celeprint µTP-100 printer, and the III–V thin-film SOA was transferred with a PDMS stamp. III–V coupons were aligned to the silicon waveguide circuit by pattern recognition. After transfer printing, oxygen plasma etching was performed to remove the photoresist, followed by a DVS-BCB full cure procedure. This approach allowed for the implementation of complex PICs on an advanced silicon optical platform.

### 3.4 Summary of integrated laser development

Table 2 summarizes the performance specifications of a selection of silicon-based integrated tunable semiconductor lasers.

**Table 2:** Performance comparison of tunable semiconductor lasers.

Structure	Integrated method	Tuning range [nm]	Maximum output power [mW]	linewidth [kHz]	SMSR [dB]
Si-SGDBR [98]	Heterogeneous integration	12	2.5	/	>30
Si-MRRS [69]	Hybrid integration	8	1.6	/	>35
Si-MRRS [71]	Hybrid integration	65	100	<15	>45
Si-MRRS [73]	Hybrid integration	99	35	/	/
Si-MRRS [74]	Hybrid integration	42.7	30	12	/
Si-MRRS [75]	Hybrid integration	55	38.5	60	>30
Si-MRRS [78]	Hybrid integration	32	/	/	/
Si-MRRS [85]	Hybrid integration	41.6	2	/	40
Si-SGDBR [41]	Heterogeneous integration	35	7.5	/	>40
Si-SGDFB [99]	Heterogeneous integration	55	0.4	/	>33
Si-MRRS [87]	Heterogeneous integration	40	3.3	338	>35
Si-MRRS [97]	Heterogeneous integration	40	1.5–3.3	<1000	>35
Si-MRRS [88]	Heterogeneous integration	54	10+	<100	> 45
Si-MRRS [91]	Heterogeneous integration	43	/	100–150	>40
Si-MRRS [92]	Heterogeneous integration	30	6.9	17.5	>55
Si-MRRS [94]	Heterogeneous integration	110	3	0.22	8
Si-MRRS [103]	Heterogeneous integration	120	/	0.14	>16
Si-MRRS [95]	Heterogeneous integration	118	15	<0.095	>50
Si-DFB array [54]	Monolithic integration	∼10	/	/	/
Si-DFB array [100]	Monolithic integration	∼40	/	/	/
Si-DFB array [101]	Monolithic integration	100	/	/	>50
Si-MRRS [102]	Monolithic integration	16	2.7	/	>45
Si-MRRS [68]	Micro-transfer printing	40	2	/	/
SiO_2_ -MRRS [104]	Hybrid integration	66	<12	<1	>50
Si_3_ N_4_ -MRRS [80]	Hybrid integration	81	13	0.29	/
Si_3_ N_4_ -MRRS [81]	Hybrid integration	>70	23	<0.04	>60
Si_3_ N_4_ -MRRS [82]	Hybrid integration	58.5	34	2.5	70
Si_3_ N_4_ -MRRS [105]	Hybrid integration	172	26.7	0.75	>40

## 4 Tunable laser linewidth narrowing

Linewidth is another important factor for tunable semiconductor lasers whereby narrow-linewidth characteristics allow for achieving better performance in many application areas including high-coherence optical communications, DWDM, high-resolution optical sensing, extremely accurate clock metering, LIDAR, and gravitational wave detection. We begin with linewidth theory, discuss how to narrow the linewidth of tunable semiconductor lasers, and then review research progress in this field.

### 4.1 Linewidth theory

The linewidth of semiconductor lasers is closely related to frequency noise. The spectral line shape of a laser is usually determined by the combination of high-frequency white quantum noise and low-frequency 1/*f* noise, usually showing a Lorentzian distribution in the tail (due to the white noise contribution), and a Gaussian distribution in the middle (due to the 1/*f* noise contribution). When only the white noise influences, the spectral lines exhibit a Lorentzian distribution, and the resulting linewidth is called the “Lorentzian linewidth”, also called the “intrinsic linewidth”.

In 1958, Schawlow and Townes [106] suggested that a laser can be viewed as a linear resonator driven by white noise, and they proved that the laser spectral lines are Lorentzian-shaped. They also derived the Schawlow–Townes linewidth equation:

(1) $$\Delta \nu =\frac{1}{2\pi {\tau^{\prime }}_{\text{p}}}=\frac{{R^{\prime }}_{\text{sp}}}{2\pi n_{\text{p}}},$$

where $R_{\text{sp}}^{\prime }$ is the spontaneous emission rate, and *n*_p_ is the total number of photons stored in the cavity.

However, Eq. (1) is not accurate when lasers are operated above the threshold current. In the 1960s, Lax reinterpreted the linewidth theory by adding a 1/2 factor as a correction to Eq. (1) [107].

(2) $$\Delta \nu =\frac{{R^{\prime }}_{\text{sp}}}{4\pi n_{\text{p}}}$$

Equation (2) considers only the effect of phase perturbation on linewidth caused directly by spontaneous emission. In 1982, for the first time, Henry [108] introduced a linewidth enhancement factor *α* related to the material properties and representing the relationship between the real and imaginary parts of the refractive index in a semiconductor laser. He also proposed the following linewidth equation:

(3) $$\Delta \nu^{\prime }=\Delta \nu \left(1+\alpha^{2}\right)=\frac{R_{\text{sp}}^{\prime }}{4\pi n_{\text{p}}}\left(1+\alpha^{2}\right).$$

In Eq. (3), the term “1” represents the noise directly induced by the photons generated by spontaneous emission in the gain region, and “*α*^2^” represents the phase noise. When the spontaneously emitted photons enter the resonant cavity, the intensity of the optical field in the cavity changes. Due to the coupling relationship between the carriers and optical field, the carriers dither to return the system into balance again. Due to the Kramers–Kronig relationship, the dithering process of the carriers causes a change in the real part of the refractive index in the resonator, which brings phase noise to the resonant optical field.

According to the derivation of Tran, Huang, and Bowers [103], Eq. (3) can be rewritten as

(4) $$\Delta \nu =\frac{\pi h\nu^{3}n_{\text{sp}}}{PQ\left(Q_{\text{E}}\right)},$$

where *h* is the Planck constant, *ν* is the frequency (Hz), *n*_sp_ is the population inversion factor, *P* is the total emitted power of the resonant cavity, and *Q*(*Q*_E_) is the loaded (external) quality factor of the laser cold cavity.

As seen from Eq. (4), the linewidth can be effectively narrowed if a passive external cavity is applied. Lasers based on external cavity structures, especially those based on MRR external cavities, have been extensively investigated due to their small size and integrability.

**Figure 9:**
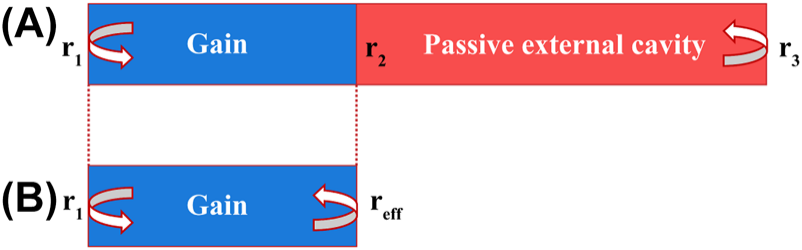
(A) External cavity model. (B) Equivalent cavity surface model.

For a typical external cavity structure, a model equivalence analysis can be conducted as shown in Figure 9. All extended cavity parts are equated as a reflecting mirror surface with complex wavelength-dependent transformation of reflectivity The linewidth equation of the laser based on the external cavity can be expressed as follows:

(5) $$\Delta \nu_{\text{external}}=\Delta \nu \frac{\left(1+\alpha^{2}\right)}{F^{2}},$$

where Δ*ν* is the corrected Schawlow–Townes linewidth, and *F* is the linewidth reduction factor, which is defined as

(6) F=1+A+B,

where *A* and *B* are given by

(7) $$A=\frac{1}{\tau_{0}}\frac{d\phi_{\text{eff}}\left(\omega \right)}{d\omega },$$

(8) $$B=\frac{\alpha }{\tau_{0}}\frac{dln|r_{\text{eff}}\left(\omega \right)|}{d\omega }.$$

Here, *τ*_0_ is the round-trip time of the photon in the active region, *ϕ*_eff_(*ω*) is the effective phase of the external cavity, and $r_{\text{eff}}\left(\omega \right)$ is the effective reflectance of the passive region. The factor *A* reflects the increase in the round-trip accumulated phase due to the extension of the effective length of the external cavity. When *A* is increased, the linewidth is reduced. The factor *B* represents the magnitude of the optical negative feedback effect, which helps stabilize the laser frequency and is proportional to *α*. In external cavity lasers, the existence of the optical negative feedback effect reduces the influence of *α* on the frequency noise, so that the laser linewidth is almost independent of *α*.

### 4.2 Research progress on linewidth narrowing

Increasing the effective cavity length of the laser is a common method for reducing linewidth. In 2015, Komljenovic et al. [88] demonstrated a tunable laser with a long external cavity. The gain section was heterogeneously integrated into the silicon waveguide via bonding and was located inside a 2 mm long cavity formed by two loop mirrors at the front and rear. Ring resonators and a phase section were controlled by thermal phase tuners. The front loop mirror (at the output of the laser) had 10% power reflection. An on-chip photoelectric detection monitor was placed at the output of the laser. The rear loop mirror had 60% power reflection, and a part of the light was coupled to the external feedback cavity. This cavity had its own phase tuning and gain sections, which were used to control the feedback level of the external cavity. The device achieved a tuning range >54 nm with a high SMSR (>45 dB). The linewidth was less than 100 kHz over the entire tuning range, and the minimum was 50 kHz.

However, as the length of the external cavity increases, the mode spacing decreases, which allows multimode excitation. By introducing structures such as MZI or high-*Q* micro-rings based on the double micro-ring structure, the linewidth can be narrowed while ensuring stable single-mode output.

In 2015, Tang et al. [74] introduced a highly asymmetric MZI based on the external cavity structure of the double micro-rings. They further increased the effective cavity length and achieved a narrow linewidth of 12 kHz and stable single-mode lasing. The optical path length of the light between the two arms of the asymmetric MZI differed significantly, thereby realizing the secondary frequency selection, improving the mode gain difference, and ensuring stable single-mode lasing. They noted that the use of highly asymmetric MZIs can achieve higher modal gain differences, narrower linewidths, and higher output powers than slightly asymmetric MZIs.

In 2017, Komljenovic et al. [91] exploited the Vernier effect of two slightly different small-sized micro-rings to realize broad tuning. In addition, they introduced a third ring with a considerably larger circumference and higher *Q* inside the laser cavity. There were two on-chip photodetection monitors in the whole device, which were located at the output end and at the high-*Q* micro-ring through-port, and were integrated into the SOI platform by bonding. With the on-chip photoelectric detection monitors, alignment of parts, such as phase, and thermal tuning of the micro-rings is performed precisely. It also makes both the laser tuning range and bias tuning predictable. The results show that a tuning range of 43 nm was obtained, with an SMSR > 40 dB and linewidth < 150 kHz.

However, because silicon has a considerable TPA coefficient (*β*_TPA_ = 5 × 10^−12^ m/W at 1550 nm), a high optical power density in the silicon waveguide generates a strong nonlinear effect, which induces higher losses. In addition, the TPA generates additional free carriers, leading to free-carrier absorption, which causes the silicon waveguide temperature to rise, and the transmission peak to tilt towards longer wavelengths due to the high thermo–optical coefficient of silicon. This results in bistability and impedes further reduction in the linewidth. Therefore, reducing the effect of TPA and free-carrier absorption, and decreasing the optical power density in the silicon waveguide is an alternative to achieve a narrow linewidth.

The optical power density can be reduced by increasing the width of the silicon waveguide; however, this easily generates multi-mode excitation. Alternatively, by changing the waveguide cross section and using shallow etched silicon waveguides, a very-low-loss silicon waveguide can be obtained and linewidth reduction can be achieved [109]. In 2020, Tran et al. [94], designed a three cascaded micro-rings structure. The three micro-ring waveguides using extremely shallow etched ridge structure with only 56 nm etched in 500 nm silicon, exhibited an ultralow loss of 0.16 dB/cm. The silicon-based integrated tunable laser can achieve an ultra-narrow Lorentzian linewidth < 220 Hz and ultrawide tuning range of 110 nm across the S + C + L band; however, the radii of the three rings are very large, being approximately 600 μm, with the resulting effect of a relatively small SMSR (approximately 8 dB). Since over-annealing is required during wafer bonding with a resulting high p-contact resistance, the gain section is damaged, which leads to low optical power output and performance degradation during testing. New processes have been developed in the frame of the heterogeneous silicon photonics platform. In 2022, Morton et al. [95] demonstrated an optimized laser which achieved 118 nm wavelength tuning range (covering S, C, and L-bands) with Lorentzian linewidth < 95 Hz, and on-chip output power of approximately 15 mW.

**Figure 10:**
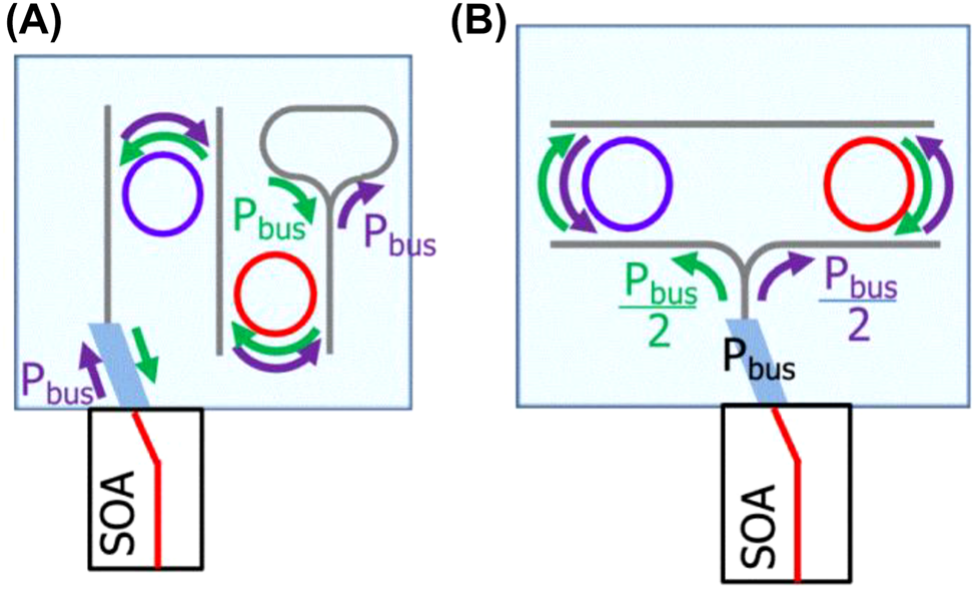
(A) Series double-loop structure. (B) Parallel double-loop structure.

In 2016, Kita et al. [75] proposed a parallel double micro-ring structure (Figure 10B) to reduce the influence of high-power and strong TPA in the cavity of the series double-ring structure (Figure 10A). The light beam was split at the Y-structured waveguide, and the optical power coupled to the ring resonator was halved. The TPA, being proportional to the square of the optical power, was reduced to one-fourth compared with that of the series structure shown in Figure 10A. Furthermore, the linewidth was narrower compared with that of the cascade structure, while high output power and stable single-mode output were obtained.

Due to the silicon material properties, the minimum achievable linewidth is limited by nonlinear effects such as TPA and free-carrier absorption, so that the linewidth cannot fall below few hundred Hz. However, silicon nitride has a wider band gap than that of silicon and is not limited by TPA effects. Therefore, tunable narrow-linewidth lasers based on a silicon nitride platform have been developed in recent years [52, 110].

In 2017, Fan et al. [80] used a structure similar to that proposed by Komljenovic [91], but based on silicon nitride waveguides, and achieved an 81 nm tuning range with a linewidth of only 290 Hz, corresponding to a reduction of nearly three orders of magnitude.

In 2020, Fan et al. added a 33 mm long spiral silicon nitride low-loss waveguide to their previous structure [80]. The silicon nitride waveguide adopted symmetrical double-stripe geometry, and the two silicon nitride core layers were separated by 500 nm. The waveguide transmission loss was less than 0.1 dB/cm. The laser achieved a tuning range >70 nm with a linewidth < 40 Hz, which is the narrowest linewidth level achieved to date.

Based on a silicon nitride waveguide, the linewidth can be further narrowed, but there are still many problems that need to be solved.

First, the thermo–optical coefficient of silicon nitride is only one-seventh that of silicon, and the power consumption of thermal tuning is higher. Second, due to the low refractive index contrast of the thin silicon nitride waveguide, the optical field confinement is low, resulting in a large amount of light leakage in the silicon dioxide cladding. Finally, the presence of a thick silicon dioxide cladding between the heating electrode and silicon nitride waveguide limits the thermal conduction rate, lowering the tuning rate. Based on this, in 2021 Guo et al. [82] proposed using a thicker (800 nm) silicon nitride waveguide to improve the above-mentioned shortcomings. The thicker silicon nitride waveguide allowed a thin oxide cladding layer, which improved the thermal tuning performance. The tunable laser output power of the Vernier double ring was 34 mW, the linewidth was only 2.5 kHz, the wavelength tuning range was 58.5 nm, and the SMSR was >70 dB. The 2*π* phase-shift tuning power of the double micro-ring was 240 mW. The rise and fall times for switching the two wavelengths were 24.1 and 60.7 μs, respectively. At modulation speeds of 10 and 20 kHz, the sweep ranges were 2.68 and 1.5 GHz, respectively. This excellent performance can already be used in frequency-modulated continuous-wave coherent LIDAR systems.

In 2022, Guo et al. [105] improved the structure of the silicon nitride external cavity chip consisting of a double MRR-based Vernier filter and a tunable Sagnac loop ring mirror, which allowed reflection index changes by tuning the phase difference between the two arms of the MZI. Due to the broadband gain spectrum, low-loss silicon nitride waveguide, and external cavity design, the laser achieved an unprecedented 172 nm tuning range. The intrinsic linewidth was <4 kHz, the SMSR was >40 dB over the entire tuning range, and the maximum output power at 1550 nm was 26.7 mW.

Research on low-loss silicon nitride waveguides allowed achieving excellent narrow-linewidth performance on silicon nitride platforms [52]. The close compatibility of the silicon nitride platform with CMOS circuits also raises the possibility of repeatable, large-scale production of complex and high-precision devices. However, the research described above is based on a hybrid integration platform, and it is difficult to realize heterogeneous integration on a silicon nitride platform. The reason is the large refractive index mismatch between silicon nitride and III–V (InP or GaAs) gain materials [111, 112] To solve this problem, a silicon interlayer can be used to achieve refractive index matching, in which case, the overall waveguide shows a III–V/Si/SiN structure [113]. This structure not only provides optical gain for silicon nitride photonic circuits, but also enriches their photonic functionality.

An alternative to reduce the linewidth is to use quantum dots as gain material. The *α* of the general quantum well laser is 2–5, whereas quantum-dot-based lasers allow significant reduction in *α* by reducing the variation in quantum-dot size and fine-tuning the p-type doping. The gain can also be reduced in the quantum-dot laser [114]. Moreover, it is possible to realize zero *α* in quantum-dot lasers. In 2020, Malik et al. [93] proposed a heterogeneous integration of widely tunable quantum-dot lasers on SOI platforms. A 52 nm tuning range and 58 dB SMSR were achieved using Vernier double micro-ringed and MZI structures. The measured Lorentzian linewidth of this quantum-dot laser was as low as 5.3 kHz.

### 4.3 Linewidth performance comparison by different integration methods

**Figure 11:**
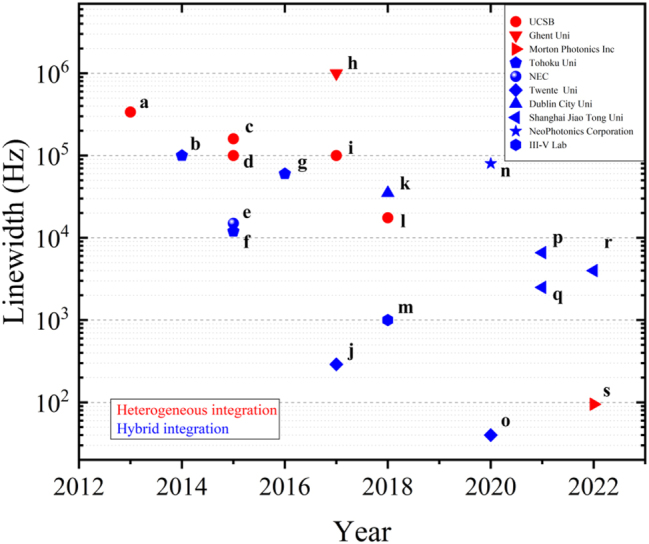
Progress of silicon-based tunable laser linewidth performance. a [87], b [72], c [89], d [88], e [71], f [74], g [75], h [97], i [91], j [80], k [115], l [92], m [104], n [116], o [81], p [83], q [82], r [105], s [95]. (The different colors represent the different integration methods. The red color represents the heterogeneous integration and the blue color represents the hybrid integration.)

Figure 11 exemplifies the performance of silicon-based integrated tunable lasers on heterogeneous integration and hybrid integration platforms in the last decade. Although the linewidth performances of lasers based on the hybrid integrated platform are presently more dominant, those of heterogeneous integrated lasers are constantly improving. Following the research and development of a heterogeneous integration platform based on silicon nitride, it is believed that the linewidth of tunable lasers based on heterogeneous integration can be further improved up to the 100 Hz level.

## 5 Future research

### 5.1 SiN platform

Although ultra-low loss silicon waveguides have been researched [117, 118], their performance is still inferior to that of silicon nitride due to TPA and free-carrier absorption(FAC). At present, research based on silicon nitride platform has been gradually carried out, and ultra-narrow linewidth tunable lasers have been realized through hybrid integration [81–83, 105], but most recent demonstrations of such devices still suffer from a large coupling loss between the III–V gain chip and the SiN edge coupler. Another approach has been proposed to use intermediate III–V structures for the index transitioning which make the heterogeneous III–V/SiN platform possible [119]. Like the silicon platform, full monolithic integration of III–V with SiN is still the most attracting method.

### 5.2 Direct growth of quantum dots on silicon

Direct growth of quantum dot (QD)-based III–V gain materials on a Si substrate is another attractive research area. The most well-developed QD material system is In(Ga)As grown on (001) GaAs or InP lattice constant materials [120] and QDs have also been proved to be less sensitive to defects than conventional quantum well (QW) structures due to effective carrier localization [121, 122], which makes epitaxially integrated lasers on silicon possible and significantly improves the manufacturing scalability [123]. Also, due to the discrete density of states and inhomogeneously broadened gain lead to QD lasers with low threshold, high temperature tolerance, ultrafast gain recovery and longer lifetime [124–126].

### 5.3 Tuning mechanism

Thermal tuning is widely used because of the low optical loss and wide tuning range, however, slow tuning speed, cross talk, and generally high-power consumption limit the application fields. Cladding materials with negative thermal optical coefficient or higher thermal conductivity can decrease the power consumption [127]. Another method is to take a different tuning mechanism. Tunable laser with essentially zero tuning power consumption has been realized through integrating a metal-oxide-semiconductor (MOS) capacitor into the laser cavity [128]. The MOS capacitor makes it possible to introduce the plasma-dispersion effect and thus change the laser modal refractive index and FCA loss to tune the laser wavelength and output power. Recently, progresses have been made in integrating the Pockels effect into a semiconductor laser. The hybrid laser integrates III–V to the lithium niobate substrate and realizes a 20 nm tunning range. Most importantly, it achieves the high frequency modulation speed of 2.0 × 10^18^ Hz/s and fast switching at 50 MHz [129].

## 6 Conclusions

In this study, we summarized and compared the research and development status of several different integration technologies, starting with silicon-based integration technology. Notably, hybrid and heterogeneous integration technologies have been widely studied and applied to silicon photonics platforms. Monolithic integration and μTP technologies are not yet mature; however, owing to their predicted large-scale integrability, low cost, and high integration density, among other excellent performance characteristics, they are expected to be the mainstream solution of integration technology in the future.

Tunable lasers have advanced over the decades and are currently evolving towards miniaturization, and integrated tunable lasers are the trend of the future. In this study, we investigated and summarized the recent developments of silicon-based tunable semiconductor lasers based on different integration methods. The linewidth has become one of the most important criteria for the performance of tunable semiconductor lasers. Narrow-linewidth wideband tunable semiconductor lasers have become the core light source for high-speed optical communication, coherent space laser communications, and coherent optical detection. In this study, starting with linewidth theory, we summarized the implementation methods and research progress of narrow-linewidth lasers. The material properties of silicon nitride make it a promising narrow-linewidth candidate that is likely to be used in future applications such as coherent optical receivers and transmitters, optical networks, and biosensing. Solving the problem of high refractive index mismatch between silicon nitride and III–V materials has become the key to realizing heterogeneous integrated narrow-linewidth tunable lasers on a silicon nitride platform.

In summary, silicon-based integrated tunable semiconductor lasers have become an essential light source in fields such as optical communications wavelength division multiplexing, LIDAR, and medical services. Further research is necessary to achieve better quality, higher yield, and cost-effective integration methods. Wider and faster tuning, and narrower linewidth lasers are also promising avenues for future research.
